# Various criteria in the evaluation of biomedical named entity recognition

**DOI:** 10.1186/1471-2105-7-92

**Published:** 2006-02-24

**Authors:** Richard Tzong-Han Tsai, Shih-Hung Wu, Wen-Chi Chou, Yu-Chun Lin, Ding He, Jieh Hsiang, Ting-Yi Sung, Wen-Lian Hsu

**Affiliations:** 1 Institute of Information Science, Academia Sinica, Nankang, Taipei 115, R.O.C, Taiwan; 2 Department of Computer Science and Information Engineering, National Taiwan University, Taipei 106, R.O.C, Taiwan; 3 Department of Computer Science and Information Engineering, Chaoyang University of Technology, Taichung County 413, R.O.C, Taiwan

## Abstract

**Background:**

Text mining in the biomedical domain is receiving increasing attention. A key component of this process is named entity recognition (NER). Generally speaking, two annotated corpora, GENIA and GENETAG, are most frequently used for training and testing biomedical named entity recognition (Bio-NER) systems. JNLPBA and BioCreAtIvE are two major Bio-NER tasks using these corpora. Both tasks take different approaches to corpus annotation and use different matching criteria to evaluate system performance. This paper details these differences and describes alternative criteria. We then examine the impact of different criteria and annotation schemes on system performance by retesting systems participated in the above two tasks.

**Results:**

To analyze the difference between JNLPBA's and BioCreAtIvE's evaluation, we conduct Experiment 1 to evaluate the top four JNLPBA systems using BioCreAtIvE's classification scheme. We then compare them with the top four BioCreAtIvE systems. Among them, three systems participated in both tasks, and each has an F-score lower on JNLPBA than on BioCreAtIvE. In Experiment 2, we apply hypothesis testing and correlation coefficient to find alternatives to BioCreAtIvE's evaluation scheme. It shows that right-match and left-match criteria have no significant difference with BioCreAtIvE. In Experiment 3, we propose a customized relaxed-match criterion that uses right match and merges JNLPBA's five NE classes into two, which achieves an F-score of 81.5%. In Experiment 4, we evaluate a range of five matching criteria from loose to strict on the top JNLPBA system and examine the percentage of false negatives. Our experiment gives the relative change in precision, recall and F-score as matching criteria are relaxed.

**Conclusion:**

In many applications, biomedical NEs could have several acceptable tags, which might just differ in their left or right boundaries. However, most corpora annotate only one of them. In our experiment, we found that right match and left match can be appropriate alternatives to JNLPBA and BioCreAtIvE's matching criteria. In addition, our relaxed-match criterion demonstrates that users can define their own relaxed criteria that correspond more realistically to their application requirements.

## Background

Biomedical named entity recognition (Bio-NER) is a fundamental technique for literature mining. It can be applied to various applications, such as disease-treatment relation extraction [1], gene list creation [2], semantic relation extraction between concepts in a molecular biology ontology [3], and gene function identification [4]. Bio-NER influences the performance of applications both in precision and recall. However, choosing an appropriate assessment method may depend on the context in which a Bio-NER text mining system is used.

In the early days, large corpora were not available and most researchers had to build small, ad-hoc corpora to evaluate their systems. The main drawbacks of such evaluations are: (1) Developers and annotators usually belong to the same group. (2) The corpora are usually not available to other researchers. (3) Only few or limited kinds of proteins and genes are annotated. (4) The corpora do not have explicit tagging guidelines so that such evaluations lack objectivity since it may be easy to design a system to fit a certain corpus; also, it is difficult to perform cross-system comparisons due to the specificities between different datasets and domains.

In recent years, the GENIA [5], GENETAG [6,7], and iProLINK [8] corpora were released. The first two are most frequently used in Bio-NER evaluation. Therefore, we describe them in detail below.

GENIA consists of 2,000 MEDLINE abstracts retrieved using the MeSH search terms *human*, *blood cell *and *transcription factor*. These abstracts are then annotated manually. Based on the GENIA ontology, the GENIA corpus classifies each biomedical NE according to its chemical structure, which is usually independent of the biological context in which it appears. Details on NE taxonomy in GENIA can be found in (Ohta et al., 2002). In GENIA, the number of NE classes is 36.

GENETAG includes a total of 20,000 sentences selected from MEDLINE abstracts. On average, one MEDLINE abstract comprises ten sentences; therefore, GENETAG amounts to about 2,000 abstracts. To ensure the heterogeneity of the GENETAG corpus, the MEDLINE sentences were first scored for term similarity to documents with known gene names. Then 10,000 high-scoring sentences and 10,000 low-scoring sentences were chosen at random. These sentences were run through AbGene [9] and manually annotated with gene and protein names by biochemistry, genetics and molecular biology experts. 15,000 of these sentences were used in the BioCreAtIvE-2004 Task 1A [6]. GENETAG has only one class per se, as it groups proteins, DNAs, and RNAs into the NEWGENE class. Based on gene names found in GenBank, this NEWGENE class includes domains, complexes, subunits and promoters (but only if they refer to a specific gene/protein) [7].

These three corpora provide relatively generous amounts of training data to perform objective evaluation metrics on machine-learning-based systems. Besides, they have explicit tagging guidelines and are freely available. As a consequence, most NER systems are evaluated on these corpora using "exact match" as the primary matching criterion. Judged according to the exact-match criterion, a candidate NE can only be counted as a match if both its boundaries and its class fully coincide with an annotated NE.

However, requiring exact matches may not be necessary in every Bio-NER application. For example, in a relation extraction application, the goal may be to determine if a sentence mentions a gene and its function. If this is done using patterns with wildcards [4], exact NE boundaries are usually unnecessary; only the existence of an NE matters.

Using exact match, we encounter another major problem with NEs whose boundaries have many variations. In real-world cases, certain NEs may be tagged in several ways, having either flexible boundaries or fitting into multiple categories (e.g., both "no correlation between serum <gene>LH</gene>" and "no correlation between <gene>serum LH</gene>" are correct in BioCreAtIvE). This problem of annotation inconsistency is intrinsic to the annotation of any corpus whether by human or machine. Inter-annotator agreement for Bio-NEs is between 87% [10] and 89% [8,11]. Inconsistencies also exist in the work of single annotators. There are several reasons for these discrepancies: in some cases, the tagging guidelines do not define how a certain phrase should be tagged; in others, multiple tagging is allowed; and in still others, human errors occur. NER systems that come across one of these irregularly tagged NEs may correctly identify a valid NE without exactly matching the corresponding human-annotated NE. Using exact match in these cases will not only generate false positives but also false negatives, effectively producing two errors where none exist – for example, simultaneously missing the target NE and tagging a partial match.

Some previous works have also addressed these problems. Seki and Mostafa [12] proposed four matching criteria in addition to exact match. They compared Kex [13] and Yapex [14] systems, which were developed in 1998. These were both rule-based systems and their evaluation dataset contained only 100 abstracts. Using different matching criteria, the authors analyzed these two systems' characteristics. Although, this evaluation was performed with rule-based systems on a small dataset, researchers can still use these matching criteria to refine a Bio-NER system or post-process the results. For example, Fukada et al. [15] calculated the core-term recognition performance in the first stage and used this to refine their system in the second stage. The JNLPBA 2004 Bio-NER task [16] included left match and right match in its evaluation tool to provide alternative perspectives on Bio-NER evaluation. The BioCreAtIvE 2004 task [6], on the other hand, allowed several possible correct annotations, of which NER systems need only match one. Their multiple-tagging scheme tags all possible meaningful boundaries of an NE and can provide more versatile assessment of Bio-NER systems. However, most annotated corpora do not adopt this annotation scheme.

In this paper, we present a comprehensive survey of commonly used matching criteria, explain their potential uses and definitions, and compare their characteristics. Then, we implement these matching criteria in the JNLPBA's evaluation tool, and re-evaluate the top four systems that took part in the JNLPBA 2004 task [16]. Our evaluation indicates that right match and left match may be appropriate alternatives to the combination of exact match and multiple tagging. Finally, we demonstrate that users can flexibly define their own relaxed criteria according to their needs.

### NER evaluation metrics

Most NER evaluation systems use precision, recall, and F-score to measure performance. Precision is the number of NEs a system correctly detected divided by the total number of NEs identified by the system. Recall is the number of NEs a system correctly detected divided by the total number of NEs contained in the input text. F-Score combines these two into a single score and is defined in the following equation:

F−score=2×precision×recallprecision+recall
 MathType@MTEF@5@5@+=feaafiart1ev1aaatCvAUfKttLearuWrP9MDH5MBPbIqV92AaeXatLxBI9gBaebbnrfifHhDYfgasaacH8akY=wiFfYdH8Gipec8Eeeu0xXdbba9frFj0=OqFfea0dXdd9vqai=hGuQ8kuc9pgc9s8qqaq=dirpe0xb9q8qiLsFr0=vr0=vr0dc8meaabaqaciaacaGaaeqabaqabeGadaaakeaacqWGgbGrcqGHsislcqWGZbWCcqWGJbWycqWGVbWBcqWGYbGCcqWGLbqzcqGH9aqpdaWcaaqaaiabikdaYiabgEna0kabdchaWjabdkhaYjabdwgaLjabdogaJjabdMgaPjabdohaZjabdMgaPjabd+gaVjabd6gaUjabgEna0kabdkhaYjabdwgaLjabdogaJjabdggaHjabdYgaSjabdYgaSbqaaiabdchaWjabdkhaYjabdwgaLjabdogaJjabdMgaPjabdohaZjabdMgaPjabd+gaVjabd6gaUjabgUcaRiabdkhaYjabdwgaLjabdogaJjabdggaHjabdYgaSjabdYgaSbaaaaa@65B5@

Since the boundaries and categories of Bio-NEs are often ambiguous, various matching criteria and class-merging strategies have been used for Bio-NER system evaluation. They are summarized below.

### Matching criteria for BioNER

One might think that only exact matches can be considered correct. However, in many applications, finding pieces of information is better than finding nothing at all. For example, in response to the question, "Alzheimer's disease is caused by mutation in which gene?" a system extracts "PS1" as opposed to "PS1 gene," then we could consider giving full marks or at least partial marks to that system. Furthermore, exact match may not reflect the true performance of a system. For example, say we need to identify a protein-protein interaction from the phrase "IL-2 activates p21ras proteins." A human expert annotates the phrase as "<protein> IL-2 </protein> interacts with the <protein> p21ras proteins </protein >." A Bio-NER system, meanwhile, comes up with the annotation: "<protein> IL-2 </protein> interacts with the <protein> p21ras </protein> proteins." If the Bio-NER system's result is compared to the human-annotated phrase, we see a boundary matching error in <protein> p21ras </protein>, where "protein" is not included in the tag. However, for a relation extraction system, this error could be acceptable, since the system has correctly identified "p21ras" as a protein, and this information is adequate to extract the relationship "IL-2 interacts with p21ras." Similarly, "the p21ras protein" or "the p21ras" could also be considered correct. In this situation, we need an alternative matching criterion other than exact match.

Other examples of inconsistent boundary tagging can often be found in annotated corpora, where one can find the same descriptive adjectives annotated as parts of following NEs in some cases but not in others. In fact, it may even be hard for biologists to decide whether descriptive adjectives such as "normal" or "activated" should be considered part of entity names. Take "human" for example. In the JNLPBA task, of the 1790 times it occurred before or at the beginning of an NE in the training data, it was annotated as a part of the NE only 110 times. But in the gold standard test data, it was included 129 times out of 130 [17]. This irregularity confuses Bio-NER systems and weakens the reliability of evaluation based on the exact-match criterion. To provide alternative evaluation perspectives, researchers have developed a variety of rules that relax matching criteria to different degrees. They are listed below alongside their definitions. In the following section we discuss their potential to assess the performance of Bio-NER systems.

### Boundary relaxation

#### Left match

If the left boundary matches exactly, the tagged NE is scored as a match. Using this rule, certain errors may be judged as correct, such as p21ras above. In these cases, the rightmost head words which represent the NE's category are skipped. This error may be acceptable in relation extraction and GO-ID assignment applications [4,18] since the category matches, and the core term is successfully identified.

#### Right match

If the right boundary matches exactly, the tagged NE is judged as correct. Applying this rule, errors due to missing or including preceding adjectives can be scored as correct. For example, in the sentence "We identified a putative STAT binding site in the promoter region of p27," regardless of whether "putative STAT" or "STAT" is tagged as a protein, it is counted as correct using right-match criterion.

#### Left/right match

If a tagged NE exactly matches either boundary of the human-annotated NE, the hit is counted as a match.

#### Partial match [12]

A detected NE is counted as correct when any fragment composing the NE is correctly detected.

#### Approximate match [19]

According to the approximate-match criterion, a tagged NE must be a substring of the human-annotated NE or vice versa. Left- and right-match criteria can be considered more restricted subsets of the approximate-match criterion.

**Figure 1:**
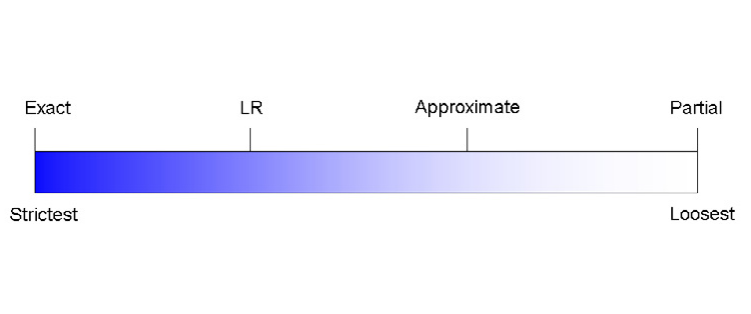
Spectrum of matching criteria.

Figure 1 shows the relationship of the above criteria from strictest to loosest. Partial match is the loosest matching criterion in the spectrum shown in Figure 1. There are two possible matching criteria that cannot be ranked on this spectrum.

#### Name part/fragment match [12]

Using fragment match, each token in an NE is considered separately. This criterion is used to assess what percentage of an NE has been correctly recognized. Using this criterion, longer terms have more weight than shorter terms. It provides an alternative method of assessment, since most other criteria only treat a hit as right or wrong and cannot measure degrees of matching.

#### Core-term match [15]

To be considered correct, machine-annotated NEs must contain a core term. Core terms identify an NE. They often have unique orthographical features, such as capital letters, numerical figures, and special symbols – for example, "SAP" from the NE "p54 SAP kinase." Some NEs are composed of a core term, head noun/phrase on the right, and adjective on the left. This criterion is useful for Bio-NER systems that extract core terms as the first step. However, because it is only possible to identify core terms by hand, few systems use this matching criterion.

#### Multiple-tagging match

The GENETAG annotation guidelines were designed to define true positive gene/protein names in terms of their specificity and semantics [7]. Each sentence in GENETAG is annotated with acceptable alternatives to the gene/protein names it contains. Such annotation scheme allows for partial matching with specificity and semantic constraints, and is a more meaningful measure of the performance of an NER system than unrestricted partial matching. For instance, the specificity constraint allows entities such as *tat DNA sequence*, but not *DNA sequence*. Semantic constraints are rules stating that the tagged entity must represent its true meaning in the context of the sentence. These constraints are geared towards multiword entities, especially ones that include numbers, letters and acronyms. For example, the name in the phrase "rabies immunoglobulin (RIG)" requires *rabies *because *RIG *implies that the gene mentioned in this sentence refers to the *rabies immunoglobulin*, and not just any *immunoglobulin*. Unlike traditional exact match, NER systems identifying any alternative tagging of an NE are scored as correct.

#### Categorical relaxation

Certain categories can be merged to reduce the ambiguity of NE classification.

#### Merging protein, DNA, and RNA

In the BioCreAtIvE Task 1A, DNA, RNA and protein names are placed in the same category. No distinctions are made between genes, proteins, RNA, domains, complexes, sequences, fusion proteins, etc. While finer-grained classification is possible, it is not really feasible in practice because even human annotators agree only 77% of the time on protein, gene and RNA classification [7]. Also, although machine learning systems can correctly categorize proteins, genes and RNAs with 78–84% accuracy [20], most Bio-NER systems do not make these distinctions because the information is not required. Hatzivassiloglou et al. [20] also found that their machine learning algorithms did not perform well against a human baseline model, suggesting that either the human model was correct, and the decreased performance was due to classification difficulty, or the machine-learning programs were penalized for being more consistent than the human model. Either way, the inclusion of these categories in the gold standard would be a significant additional source of ambiguity.

#### Merging cell line and cell type

Due to the large number of inconsistent or ambiguous annotations of these two categories in biomedical corpora, combining "cell type" and "cell line" into the single class "cell" can effectively reduce the number of annotation errors detected. In addition, in some biomedical applications, such as semantic role labelling, we only want to extract the location of molecular events in a text, no matter whether they are in vitro (cell line) or in vivo (cell type).

## Results and discussion

### Experiment Design

In our experiments, we aim to examine the impact of different tagging constraints on the measurement of system performance. We conduct four experiments described below.

Experiment 1 examines the impact of multiple tagging. We compare the performance of systems that participated in both the JNLPBA task and the BioCreAtIvE task by simulating the BioCreAtIvE classification scheme on the JNLPBA dataset. In BioCreAtIvE, no distinctions are made between genes, proteins, RNA, domains, complexes, sequences, fusion proteins, etc [7]. We re-annotate protein, DNA and RNA in the JNLPBA dataset as NEWGENE. Then we run the best four JNLPBA systems (Zho [21], Fin [17], Set [22], and Son [23]), and the best four BioCreAtIvE systems (Zho [24], Fin [25], Mcd [26], and Son [27]), on the two datasets. Of these systems, three (Zho, Fin, and Son) took part in both tasks, while two (Set and Per) entered only one task each but used similar features and the same CRF package – Set BioCreAtIvE and Per JNLPBA. We use the exact match criterion for system performance evaluation.

Since most annotated corpora do not adopt the multiple-tagging approach used in BioCreAtIvE, in Experiment 2, we test a range of other matching criteria comparable to multiple tagging. We randomly sample five subsets from JNLPBA's test set (with the same modification as before), each containing 3,000 sentences. The experiment is conducted on the top four Bio-NER systems in the JNLPBA task. Seven matching criteria are compared, i.e., exact, left, right, left/right, partial, approximate, and fragment match.

Experiment 3 aims to demonstrate how users can customize assessment of Bio-NER to suit their specific applications. Consider the following scenario. A biologist may need to refer to all related macromolecules (including protein, DNA, and RNA) of a cell, when reading a biomedical article. In this application, there is no need to distinguish among protein, DNA, and RNA. In addition, it is quite difficult to distinguish cell line from cell type. For example, in the JNLPBA test set, "T cell" is classified as cell line 55 times while 237 times as cell type. Therefore, we propose the relaxed match criterion that merges protein, DNA, and RNA classes into macromolecule [28], and cell-line and cell-type into cell. Furthermore, the annotation of the left boundary tend to be more inconsistent due to the ambiguity of including the left boundary words as part of an NE. For example, "normal" is tagged as the left boundary word of an NE 34 times while 9 times not in the JNLPBA test set. As a result, we choose the right match in our relaxed match. We conduct an additional experiment on the same four Bio-NER systems as in Experiment 1.

In Experiment 4, we test five basic matching criteria from the strictest to the loosest – exact, left/right, approximate, partial, and uncategorized partial – on the JNLPBA corpus by using the top 2004 JNLPBA system, Zhou et al.'s [21] in order to find the percentage of false negatives.

### Experiment 1

The results are reported in Table 3 and Table 4, which show the precision rates, recall rates, and F-scores for NEWGENE on the JNLPBA and BioCreAtIvE datasets.

**Table 1:** Basic statistics of the JNLPBA dataset

	# abstracts	# sentences	# words
Training Set	2,000	18,546	472,006 (236.00/abs) (22.97/sen)
Test Set	404	3,856	96,780 (239.55/abs) (22.72/sen)

**Table 2:** Absolute (and relative) frequencies of all NE classes in each part of the JNLPBA dataset

	Protein	DNA	RNA	Cell Type	Cell Line	All
Training Set	30,269 (59.0)	9,533 (18.6)	951 (1.9)	6,718 (13.1)	3,830 (7.5)	51,301 (100)
Test Set	5,067 (58.5)	1,056 (12.2)	118 (1.4)	1,921 (22.2)	500 (5.8)	8,662 (100)

**Table 3:** Performance on the JNLPBA dataset with protein, DNA, and RNA merged into one category

	Zho [21]	Fin [17]	Set [22]	Son [23]
Precision (%)	70.92	70.93	70.71	67.26
Recall (%)	80.56	77.96	76.36	74.09
F-score (%)	75.43	74.28	73.43	70.51

**Table 4:** Performance on the BioCreAtIvE dataset

	Zho [24]	Fin [25]	Mcd [26]	Son [27]
Precision (%)	82.00	79.20	86.40	80.00
Recall (%)	83.17	85.40	78.70	68.50
F-score (%)	82.58	82.20	82.40	73.80

Using the exact-match criterion, F-scores are lower on the JNLPBA than on BioCreAtIvE. We believe that the disparity in performance between the two is due to our evaluation system's lack of alternative-tagging rules, which the original BioCreAtIvE task employs. The other reason is that, though our modification of the JNLPBA task's evaluation system using BioCreAtIvE's unified protein/DNA/RNA class improved scores, it is not a perfect re-creation of BioCreAtIvE's NEWGENE class, because the two tasks' original class definitions differ. Since some users may need BioCreAtIvE's evaluation scheme but do not have the same annotation scheme as BioCreAtIvE in their corpora, we are looking for a matching criterion that can be an appropriate alternative to BioCreAtIvE's method.

### Experiment 2

To find the closest matching criterion to BioCreAtIvE's, we apply two statistical methods: hypothesis testing and correlation coefficient. For each matching criterion, we test if its hypothesis (*H*_*0*_) – whether its average F-score is equal to BioCreAtIvE's – can be accepted at confidence level α = 0.05. For hypothesis testing, we perform the third experiment. Since the experiment is conducted on four Bio-NER systems and each system runs on five datasets, we end up with 20 samples from the JNLPBA dataset. In Table 5, we show the evaluation results for all seven matching criteria ("J-" stands for evaluations carried out on the JNLPBA dataset). Only the left-match and right-match criteria pass this hypothesis test. Right match, which has the largest correlation coefficient between its F-score and the F-score evaluated on BioCreAtIvE (Table 6), is, therefore, the criterion closest to BioCreAtIvE's multiple tagging method.

**Table 5:** Hypothesis testing on the equivalence of each matching criterion to BioCreAtIvE's multiple-tagging scheme

	X¯ MathType@MTEF@5@5@+=feaafiart1ev1aaatCvAUfKttLearuWrP9MDH5MBPbIqV92AaeXatLxBI9gBaebbnrfifHhDYfgasaacH8akY=wiFfYdH8Gipec8Eeeu0xXdbba9frFj0=OqFfea0dXdd9vqai=hGuQ8kuc9pgc9s8qqaq=dirpe0xb9q8qiLsFr0=vr0=vr0dc8meaabaqaciaacaGaaeqabaqabeGadaaakeaacuWGybawgaqeaaaa@2DFD@ (%)	S^ MathType@MTEF@5@5@+=feaafiart1ev1aaatCvAUfKttLearuWrP9MDH5MBPbIqV92AaeXatLxBI9gBaebbnrfifHhDYfgasaacH8akY=wiFfYdH8Gipec8Eeeu0xXdbba9frFj0=OqFfea0dXdd9vqai=hGuQ8kuc9pgc9s8qqaq=dirpe0xb9q8qiLsFr0=vr0=vr0dc8meaabaqaciaacaGaaeqabaqabeGadaaakeaacuWGtbWugaqcaaaa@2DEB@ (%)	*H*_0_	*t*_0_(%)	Accept *H*_0_?*
J-Exact	74.20	1.92	M = 80.25%	-14.07	No
J-Left/Right	84.19	1.17	M = 80.25%	15.01	No
J-Approximate	85.76	1.20	M = 80.25%	20.59	No
J-Partial	85.92	1.16	M = 80.25%	21.94	No
J-Left	79.72	1.20	M = 80.25%	-1.95	Yes
J-Right	80.87	1.60	M = 80.25%	1.75	Yes
J-Fragment	83.83	1.82	M = 80.25%	8.81	No

*the condition for accepting *H*_0 _is *t*_(0.025,*v *= 19) _≤ *t*^0 ^≤ *t*_(0.975,*v *= 19)_, where *t*_(0.025,*v *= 19) _= -2.093 and *t*_(0.975,*v *= 19) _= 2.093.

**Table 6:** Correlation coefficient of each matching criterion with BioCreAtIvE

	Zho	Fin	Set/Mcd	Son	Correlation coefficient
BioCreAtIvE	82.58%	82.20%	82.40%	73.80%	-
J-Exact	75.43%	74.28%	73.43%	70.51%	0.9286
J-Left/Right	83.75%	84.88%	84.46%	82.73%	0.8491
J-Approximate	84.88%	86.68%	86.34%	85.24%	0.3892
J-Partial	85.01%	86.74%	86.53%	85.46%	0.3476
J-Left	80.01%	79.97%	79.42%	77.69%	0.9688
J-Right	80.89%	81.53%	81.10%	78.05%	0.9788
J-Fragment	85.47%	84.41%	83.51%	81.44%	0.8926

It can be observed from Table 6 that left match is second to right match by only a slight margin. This finding can be explained by the following observation: While most NEs have head nouns either on their right or left boundaries, more have them on the right. Right match and left match are both potential alternatives to BioCreAtIvE's multiple-tagging method. If we want to avoid overestimating performance of systems that are only adept at tagging right boundaries, we can simultaneously double check using left or exact match. It is also worth mentioning that left/right match is inferior to both right match and left match in terms of hypothesis testing results and correlation coefficient. This may imply that boundary conditions can only be loosened to a certain extent.

### Experiment 3

We compare the best systems' performance evaluated using the traditional five-class exact-match criterion and the proposed relaxed-match criterion. The results are shown in Table 7, where we only report the best rates among the four systems. Using the relaxed-match criterion, the best Bio-NER system can achieve 77.9%, 85.6%, 81.5% in precision, recall, and F-score respectively, which more realistically reflect the performance of this specific application.

**Table 7:** Comparison of the best results using exact and relaxed evaluation

Evaluation criterion	NE categories	Matching criterion	# of NE classes	Best system	Best performance(%)		
					
					Precision	Recall	F-score
Exact	protein, DNA, RNA, cell line, cell type	exact match	5	Zho [22]	69.4	76.0	72.6
Relaxed	macromolecule (Protein + DNA + RNA), Cell (cell line + cell type)	right match	2	Fin [15]	77.9	85.6	81.5

### Experiment 4

The results are reported in Table 8, which shows the precision rates, recall rates and F-scores that the best JNLPBA participant system achieved under five basic matching criteria – exact, left/right, approximate, partial, and uncategorized partial – as described at the beginning of the Methods Section. The maximum recall rates are 76.0% for exact, 83.2% for left/right, 84.8% for approximate, and 85.3% for partial match. The last column reports the performance gains achieved by ignoring all NE classification. The maximum recall rate of 91.7% represents a gain of 6.4% over partial match. The remaining 8.3% represents NEs that have been completely missed. Of course, if no matching parts exist, it is impossible for post-processing to fix the boundary errors by extending core terms [15]. According to our analysis, many complete-miss errors were due to inconsistent annotation in the JNLPBA 2004 training data, especially untagged cell-line NEs. We found that many instances of "T cell," "Peripheral blood neutrophil," and "NK cell" were not tagged as cell line. This inconsistency confuses machine learning algorithms, leading to a large number of false negatives. How to uncover false negatives remains a challenging issue in Bio-NER.

**Table 8:** Results of the best JNLPBA participant system under different matching criteria

	Exact	Left/Right	Approximate	Partial	Uncategorized Partial
Precision (%)	69.4	77.0	77.3	77.4	83.9
Recall (%)	76.0	83.2	84.8	85.3	91.7
F-score (%)	72.6	80.0	80.8	81.2	87.7

## Conclusion

We present a survey of commonly used matching criteria, explain their potential uses and definitions, and compare their characteristics. We also compare two popular Bio-NER evaluation methods – those used by BioCreAtIvE and the JNLPBA. From our statistical tests (as shown in Tables 6 and 7), we find that right match has no significant difference and has the highest correlation coefficient with BioCreAtIvE's multiple-tagging criterion. In addition, left match is also comparable to BioCreAtIvE's multiple-tagging criterion though slightly inferior to right match. In biomedical applications where strict exact-boundary match is not necessary, right or left match may be sufficient and useful. Researchers can use both criteria to evaluate Bio-NER systems and use the results for further analysis to improve the systems. Our study shows that, evaluated with the relaxed match, the best system's performance is above 80%. Users can flexibly define their own relaxed criterion according to their application context.

## Methods

### Experimental Datasets

In this paper, we compare several Bio-NER systems' performance on two corpora. The first is the corpus of BioCreAtIvE 2004 Task 1A, which is adopted from the GENETAG corpus (discussed in the Background section). The second is the corpus of the JNLPBA 2004 shared task, which is derived from the GENIA corpus. Note that the BioCreAtIvE corpus provides multiple tagging (discussed in the Methods section) and the JNLPBA does not.

GENETAG is a heterogeneous set of sentences that contain many true positive gene names, and also many non-gene entities that are morphologically similar to gene names. There are approximately 24,000 instances of gene/protein names in the 20 K sentences. 15,000 of the sentences were used in the BioCreAtIvE-2004 Task 1A [6]. GENETAG annotation guidelines were designed to define real gene/protein names in terms of their specificity and semantics. Each gene/protein name in GENETAG is annotated with all acceptable alternatives.

In the JNLPBA 2004 shared task, the GENIA corpus is used as training data. However, the original 36 classes are simplified to 5 classes: protein, DNA, RNA, cell line and cell type (See Table 1 for detailed statistics). To simplify the annotation task to a linear sequential analysis problem, embedded NEs have been removed leaving only the outermost NEs. Coordinated NEs with ellipses are annotated as one NE as in the following example:

... in [lymphocytes] and [T- and B- lymphocyte] count in ...

Here, "T- and B- lymphocyte" is annotated as a single NE although it includes two entity names. Similarly, "lymphocytes" is annotated as one NE though it is plural.

The test set consists of 404 newly annotated MEDLINE abstracts from the GENIA project. These abstracts were annotated with the same five categories. Half of these abstracts are from the same domain as the training data and the other half are from the super-domain of "blood cells" and "transcription factors." The basic statistics for the training and test data are summarized in Table 2.

## Authors' contributions

TH Tsai designed all the experiments. SH Wu and J Hsiang discussed and refined this paper. YC Lin wrote the evaluation programs for all inexact matching criteria. WC Chou and D He, two biologists in our lab, provided biomedical background knowledge and examined Bio-NER systems' results. TY Sung and WL Hsu guided the whole project.
